# One-Step Derivation of Mesenchymal Stem Cell (MSC)-Like Cells from Human Pluripotent Stem Cells on a Fibrillar Collagen Coating

**DOI:** 10.1371/journal.pone.0033225

**Published:** 2012-03-21

**Authors:** Yongxing Liu, A. Jon Goldberg, James E. Dennis, Gloria A. Gronowicz, Liisa T. Kuhn

**Affiliations:** 1 Center for Biomaterials, Department of Reconstructive Sciences, University of Connecticut Health Center, Farmington, Connecticut, United States of America; 2 Benaroya Research Institute at Virginia Mason, Seattle, Washington, United States of America; 3 Department of Surgery, University of Connecticut Health Center, Farmington, Connecticut, United States of America; University of Reading, United Kingdom

## Abstract

Controlled differentiation of human embryonic stem cells (hESCs) and induced pluripotent stem cells (iPSCs) into cells that resemble adult mesenchymal stem cells (MSCs) is an attractive approach to obtain a readily available source of progenitor cells for tissue engineering. The present study reports a new method to rapidly derive MSC-like cells from hESCs and hiPSCs, in one step, based on culturing the cells on thin, fibrillar, type I collagen coatings that mimic the structure of physiological collagen. Human H9 ESCs and HDFa-YK26 iPSCs were singly dissociated in the presence of ROCK inhibitor Y-27632, plated onto fibrillar collagen coated plates and cultured in alpha minimum essential medium (alpha-MEM) supplemented with 10% fetal bovine serum, 50 uM magnesium L-ascorbic acid phosphate and 100 nM dexamethasone. While fewer cells attached on the collagen surface initially than standard tissue culture plastic, after culturing for 10 days, resilient colonies of homogenous spindle-shaped cells were obtained. Flow cytometric analysis showed that a high percentage of the derived cells expressed typical MSC surface markers including CD73, CD90, CD105, CD146 and CD166 and were negative as expected for hematopoietic markers CD34 and CD45. The MSC-like cells derived from pluripotent cells were successfully differentiated *in vitro* into three different lineages: osteogenic, chondrogenic, and adipogenic. Both H9 hES and YK26 iPS cells displayed similar morphological changes during the derivation process and yielded MSC-like cells with similar properties. In conclusion, this study demonstrates that bioimimetic, fibrillar, type I collagen coatings applied to cell culture plates can be used to guide a rapid, efficient derivation of MSC-like cells from both human ES and iPS cells.

## Introduction

Human embryonic stem cells (hESCs) and induced pluripotent stem cells (hiPSCs) are attractive stem cell sources for cell therapy [1], [2]. Multi-potent adult stem cells, such as human bone marrow derived mesenchymal stem cells (MSCs) show promise for the treatment of large and severe skeletal defects including repair of damaged cartilage [3], but they are limited in number and quickly lose their differentiation potential during expansion [4]. Differentiating hESCs and hiPSCs into multi-potent progenitors or overtly differentiated cells prior to transplantation is one of the most promising approaches for the safe and effective use of pluripotent stem cells. Transplantation of lineage-committed cells can avert *in vivo* teratoma formation that is caused by the rapid growth and uncontrolled spontaneous differentiation of pluripotent stem cells [5]. However, stable and efficient differentiation of hESCs and hiPSCs into the clinically relevant progenitor or mature cell types remains a major challenge.

Strategies to derive MSC or MSC-like cells from hESCs have been explored by several research groups and range from co-culture with the desired cell type [6], to supplementation of the culture medium with a cocktail of growth factors [7]. Uncontrolled spontaneous differentiation in embryoid bodies followed by flow cytometry sorting to obtain the desired phenotype has also been employed to obtain MSCs [8]. In other studies, MSCs have been obtained from spontaneously differentiating embryoid bodies (EBs) or aggregates in simple culture medium without complex growth factor supplements, although removal of the EBs and prolonged serial passaging was required [8], [9]. The cells derived by all of these methods tested positive for established MSC surface markers and were able to differentiate into two or three mesenchymal lineages *in vitro*, i.e., osteoblastic, chondrocytic, and adipocytic. While promising in many aspects, these methods have intrinsic limitations including complicated cell sorting, extensive culture time and related labor cost, and, most importantly, low efficiency and yields.

For routine clinical applications, it is necessary to develop effective, reliable, cost- and labor-efficient methods to derive MSCs or MSC-like cells from both hESCs and hiPSCs. Besides the addition of cytokines and growth factors, the use of appropriate biomaterial matrices or culture surfaces offers another means to influence cell fate through physico-chemical stimulation [10], [11] and associated signaling modulation [12], [13]. Collagen type I, a traditional biomaterial, has long been known to promote osteogenic differentiation of mesenchymal stem cells through an integrin-mediated signaling pathway [14]–[16]. It also is known for activating an epithelial-to-mesenchymal transition (EMT) of epithelial cells [17]–[19] and EMT has been used successfully by others for generation of MSC-like cells from hESCs [7]. Thus, we postulated that a collagen matrix could potentially play a positive role in regulating the differentiation of hESCs and hiPSCs towards a multi-potent mesenchymal progenitor cell.

In the present study hESCs and hiPSCs underwent differentiation while cultured on type I collagen coatings that were prepared using an improved, highly reproducible coating method characterized by the formation of a thin layer of supramolecular collagen fibrils [20], [21]. The thin coating allows excellent visibility of cellular structures and cell-matrix interactions, and provides a physiologically correct fibrillar collagen substrate. In contrast, the traditional, thick, non-fibrillar collagen coatings prepared using standard methods are limited by the inconsistency of the coating thickness, easy coating detachment, and poor visibility of the cells within the thick coating [16], [22], [23]. Multi-potentiality was assessed through tri-lineage differentiation assays (i.e. osteogenic, chondrogenic and adipogenic differentiation).

Additionally, we hypothesized that plating dissociated cells versus colonies of hESCs or embryoid bodies would also significantly increase the homogeneity of differentiation to MSC-like cells. Plating multi-cell colonies is the established method for passaging or differentiating hESCs and hiPSCs, yet plating singly dissociated cells can significantly increase the differentiation yield, as shown with osteogenic differentiation cultures [24]. Accordingly, we used singly dissociated hESCs and hiPSCs to promote the derivation of MSC-like cells. However, dissociating hESCs and hiPSCs by the traditional enzymatic methods generally results in cell survival rates usually lower than 1% [24]; therefore, two recently reported measures were taken to promote the survival of the dissociated cells in the present study. First, we pre-treated the cell clusters with ROCK inhibitor Y-27632 [25] and secondly, we used the milder enzyme medium Accutase® [26]. With this combination, singly dissociated human pluripotent cells survived well and thus the derivation of MSC-like cells from pluripotent cells in a mono-layer culture on a fibrillar collagen coating could be conducted.

## Results

### Derivation of MSC-like cells

**Figure 1:**
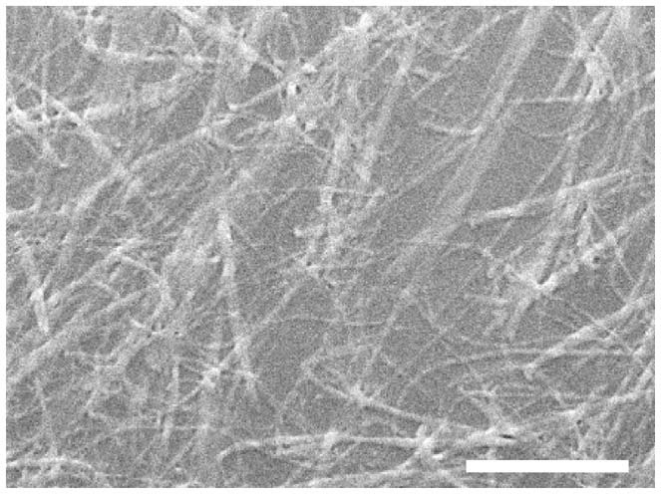
Self-assembly of Type I collagen on a tissue culture plastic substrate leads to a thin fibrillar coating. Scanning electron microscopy of the bioimimetic fibrillar type I collagen coating shown prior to cell seeding (scale bar: 5 µm).

**Figure 2:**
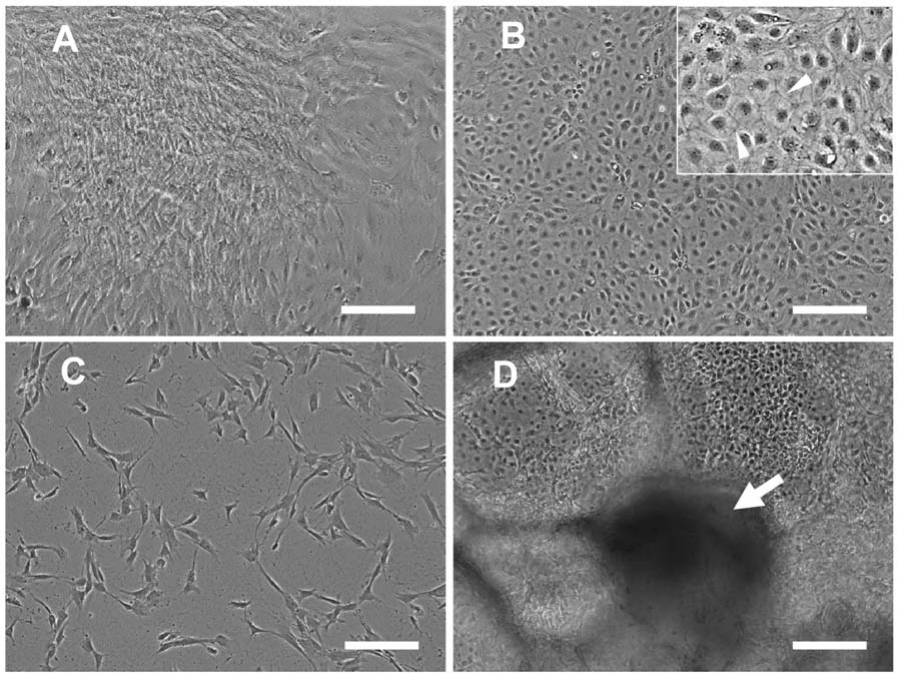
Various cell morphologies were observed in response to the cell culture plate coatings. Phase contrast images of cell morphology as a function of cell culture plate coating chemistry. Note that all the images were taken from H9-hESC cultures, but are also representative for the corresponding YK26-iPSCs cultures. (A) The typical fibroblast-like morphology of cells obtained on the collagen coating after 10 d (P0). The cells are located in separate colonies and some areas of the plate remain open. (B) The morphology of the highly confluent cells obtained on tissue culture treated polystyrene after 10 days. The inset shows an enlarged region (2×) of the image; arrowheads indicate the tight junction-like structure formed between cells. (C) The clear fibroblast-like morphology of cells subcultured (P1) on collagen coating for 4 days. (D) The varied morphology of cells derived for 10 days on either tissue culture treated polystyrene or collagen coatings after plating cell colonies rather than single cells; the arrow indicates the protrusion growing up into the medium. Scale bars for phase contrast images: 0.2 mm.

The collagen films used as substrates for the cell culture studies have a fibrillar structure as seen by scanning electron microscopy (Fig. 1). At one day after plating, the singly dissociated hESCs and hiPSCs were uniformly attached to the collagen coating. At 10 days after plating, however, only several isolated colonies remained in the cultures on the collagen coating and the colonies were predominantly located at the edge of the culture wells. Most of the interior region of the culture well was devoid of cells. The cells within the colonies on collagen had a spindle-like morphology (Fig. 2A). In contrast, the cells on the control surface of tissue culture treated polystyrene attached and grew more robustly, and formed a tight monolayer throughout the well by day 10 (Fig. 2B). The cells on tissue culture polystyrene did not have a spindle-shaped morphology, and instead featured tight junction-like structures characteristic of epithelial cells (Fig. 2B inset). After the cells cultured on the collagen coatings were trypsinized and sub-cultured onto new collagen films, they had a uniform fibroblastic-like morphology (Fig. 2C). H9-hESCs and YK26-iPSCs demonstrated similar substrate dependent attachment and morphological changes during the derivation process with more cells attaching and growing more robustly on tissue culture plastic. Repeated passaging of the cells on tissue culture polystyrene did not cause any change in morphology – the cells remained tightly packed with an epithelial morphology.

Plating the pluripotent cells as colonies, which is the common method for passaging hESCs and iPSCs, did not induce the cells to attain a spindle-shape morphology on either the collagen coating or the control tissue culture plate in this medium. A representative image of the 10 day cultures of H9-hESCs plated as colonies are shown Fig. 2D. A mixture of cell types with various morphologies growing in monolayers in some areas while also forming multi-cellular macrostructures protruding up into the medium was observed in the cultures plated as colonies. This occurred for both H9-hESCs and the YK26-iPSCs. We interpret these varied morphologies as a signature of uncontrolled spontaneous differentiation into multiple cell types that should be avoided when generating a homogeneous progenitor population.

**Figure 3:**
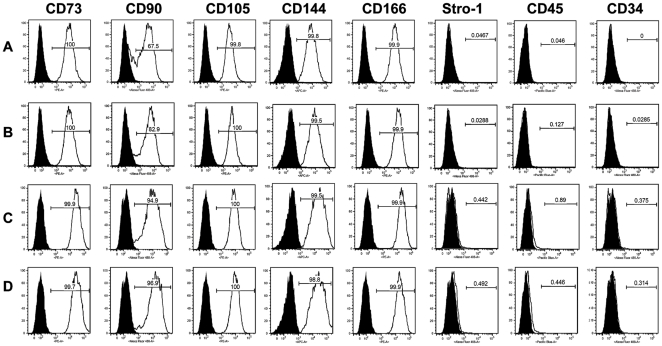
Flow cytometry analysis of surface markers consistent with adult MSCs. Surface antigen profile analysis by flow cytometry for the derived MSC-like cells. (A) and (C) are from cells derived from H9 at passage 2 and 4, respectively. (B) and (D) are from cells derived from YK26-iPSCs at passage 2 and 4, respectively. The black histogram represents the unstained control and white overlay represents each antigen. The numeric values shown within the histograms represent the percentage of the positive cells.

Flow cytometry was performed on the pluripotent cells that were differentiated through the process of being passaged twice on the collagen coating. The antigens CD73, CD90, CD105, CD146 and CD166 known to be present on adult marrow derived MSCs [9] were expressed by a high percentage of the differentiated pluripotent stem cell populations (Fig. 3). The hematopoietic markers CD34 and CD45 were detected at levels below 1% (Fig. 3) as expected for MSCs. The expression of CD90 was low relative to the other markers at passage two, 67.5% and 82.9%, for the H9 and iPS-YK26, respectively. However, after 4 passages, the expression of CD90 increased to 94.9% and 96.9% for H9-ESCs and YK26-iPSCs, respectively. Because the cells derived on collagen express a surface antigen profile similar to human bone marrow derived MSCs and have a fibroblastic morphology similar to MSCs, we refer to them as MSC-like cells. Note that the MSC-like cells derived from both the H9-hECs and YK26-iPSCs shared a very similar surface antigen profile.

### Osteogenic differentiation

**Figure 4:**
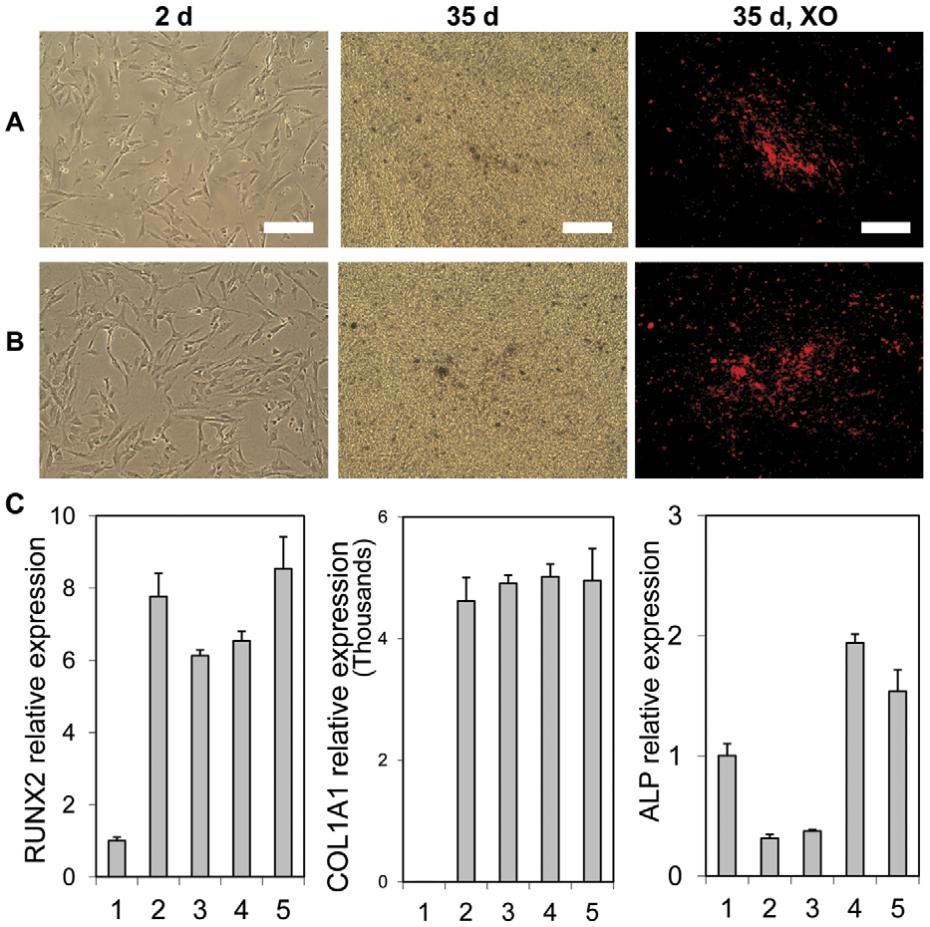
*In vitro* osteogenic differentiation. Phase contrast images of the cells in the osteogenic cultures on day 2 and 35. (A) and (B) show MSC-like cells derived from H9-hESC and YK26-iPSCs, respectively. The 35 day cultures were stained with xylenol orange dye (XO) to reveal mineral deposition. The red fluorescence in the last column of images displays the positive XO staining. Scale bars: 0.2 mm. (C) RT-qPCR analysis for the mRNA expression of osteogenic-related genes including RUNX2, COL1A1 and ALP on day 35. 1: undifferentiated H9-hESC; 2 and 3: MSC-like cells that derived from H9-hESC and YK26-iPS cells, respectively, before osteogenic differentiation; 4 and 5: 35 day osteogenic cultures of the MSC-like cells derived from H9-hESC and YK26-iPS cells, respectively.

Evidence of the multi-potentiality of the derived cells was assessed through tri-lineage differentiation experiments including osteogenic, chondrogenic and adipogenic. The MSC-like cells derived on fibrillar collagen attached and grew robustly when passaged onto tissue culture treated plates for the osteogenic differentiation studies. There was no obvious difference in attachment or growth between the cells derived from H9-hESCs or YK26-iPSCs (Fig. 4A,B). After 35 days in osteogenic medium, mineral deposition was detected in both cultures by xylenol orange (XO) staining (Fig. 4A,B). No XO staining was found in undifferentiated H9-hESCs and YK26-iPSC cells cultured under identical osteogenic conditions. The positive XO staining indicates the presence of calcium-containing mineral deposition in the cultures which is expected during osteogenic differentiation. Gene expression of the MSC-like cells was assessed before and after osteogenic differentiation. In comparison to the undifferentiated hESCs and hiPSCs, the MSC-like cells before exposure to osteogenic medium showed significantly greater expression of RUNX2 and COL1A1 genes, but not alkaline phosphatase (ALP) gene (Fig. 4C). Initially, the ALP gene expression was 1.5–2 fold less than that of hESCs and hiPSCs which is expected since ALP expression is very high in undifferentiated pluripotent cells. After 35 days of exposure to osteogenic medium, ALP expression was up-regulated by 4–6 fold in MSC-like cells, while RUNX2 and COL1A1 were maintained at significantly high levels (Fig. 4C). ALP expression is generally regarded as an early indicator of osteogenic differentiation. Osteocalcin (OC) and bone sialoprotein (BSP) gene expression was not detected, which are markers for late stage matrix-producing osteoblasts indicating the cells did not progress completely to mature osteoblasts under these culture conditions.

### Chondrogenic differentiation

**Figure 5:**
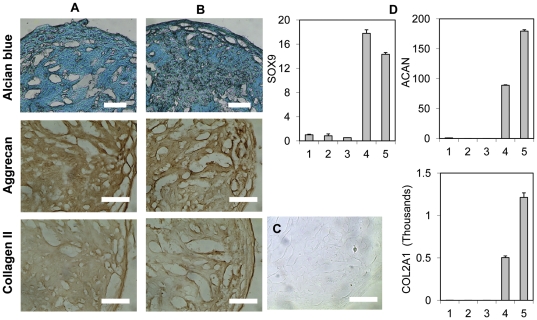
*In vitro* chondrogenic differentiation. Positive chondrogenic matrix staining observed after chondrogenic differentiation. (A) and (B) are images of 21d cultures of MSC-like cells derived from H9-hESC and YK26-iPSCs, respectively. Alcian blue staining of the histological sections of the chondrogenic pellet cultures was positive. Immunohistochemical staining for aggrecan and collagen Type II on the histological sections of the 21 d chondrogenic pellet cultures was positive. Scale bars: 0.2 mm. (C) Negative control for Col II. (D) RT-qPCR analysis for mRNA expression of chondrogenic genes including SOX9, COL2A1 and aggrecan (ACAN). 1: undifferentiated H9-hESC; 2 and 3: MSC-like cells that derived from H9-hESC and YK26-iPS cells, respectively, before chondrogenic differentiation; 4 and 5: 21 day chondrogenic cultures of the MSC-like cells derived from H9-hESC and YK26-iPS cells, respectively.

The multi-lineage potential of the MSC-like cells derived from pluripotent cells was further assayed in a chondrogenic differentiation assay performed in pellet cultures. After 21 days of culturing in chondrogenic medium, a cartilage-like glycosaminoglycan-rich matrix which stained positively with alcian blue was detected throughout the histological sections of the pellet (Fig. 5A,B). Since the cells are cultured in pellets, individual cells are not clearly visualized in the multi-cellular pellet sections. To further confirm that both cell types formed a cartilaginous matrix, the sections were immunochemically stained for aggrecan and collagen type II proteins. Both molecules were prevalent throughout the sections of both cultures (Fig. 5A,B). No immunostaining was detected in the negative controls (Fig. 5C). At 21 days, expression of SOX9, COL2A1 and ACAN genes was significantly up-regulated in pellet cultures (Fig. 5D). SOX9 was present in low amounts in undifferentiated hESCs and the MSC-like cells before the chondrogenic differentiation protocol. COL2A1 and aggrecan (ACAN) genes were not detected in the undifferentiated hESCs or in the MSC-like cells before exposure to the chondrogenic differentiation medium. The expression of SOX9 gene in the pellet cultures increased by 14–18 fold compared to the undifferentiated MSC-like cells derived from both H9 and YK26-iPSCs.

### Adipogenic differentiation

**Figure 6:**
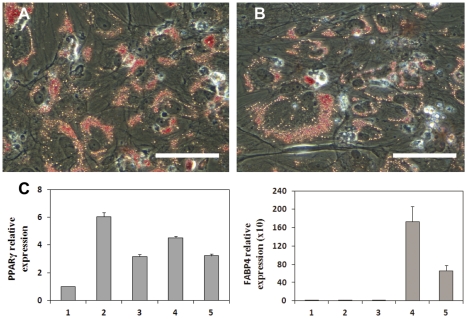
*In vitro* adipogenic differentiation. Oil red O staining of the 21 day adipogenic cultures was positive in both (A) MSC-like cells derived from H9-hESC and (B) MSC-like cells derived from YK26-iPS cells. Scale bars: 0.1 mm. (C) RT-qPCR analysis for mRNA expression of adipogenic genes PPARγ and FABP4. 1: undifferentiated H9-hESC; 2 and 3: MSC-like cells that derived from H9-hESC and YK26-iPS cells, respectively, before adipogenic differentiation; 4 and 5: 21 day adipogenic cultures of the MSC-like cells derived from H9-hESC and YK26-iPS cells, respectively.

Adipogenic differentiation was conducted on tissue culture plates. In response to the insulin stimulation, the MSC-like cells displayed significant lipid accumulation in the cytoplasm which was positively stained with Oil Red O (Fig. 6A,B). The upregulation of the expression of the FABP4 gene further confirmed adipogenic differentiation capability (Fig. 6C). PPARγ was upregulated relative to undifferentiated hESCs, but not enhanced by the adipogenic differentiation protocol. No obvious difference was noticed between cultures derived from H9 hESCs and YK26 iPSCs.

## Discussion

Unlike previously reported methods which typically rely on the addition of soluble factors to affect pluripotent stem cell differentiation, the present study reports an alternative approach using a biomaterial coating on a cell culture plate made of fibrillar collagen Type I to promote the derivation of MSC-like cells. Both hESCs and hiPSCs were subjected to MSC derivation on a fibrillar type I collagen coating prepared by self-assembly from collagen solutions on non-treated tissue culture plastic which thereby reproduces physiological Type I collagen, i.e. collagen with individual fibrils 200–300 nm in diameter and several µm in length [27]. In the present study, homogenous spindle-shaped cells were obtained after 10 days in the absence of complex growth factors, cytokines or complicated manipulation of the cultures when grown on biomimetic fibrillar collagen. The present method successfully avoided the necessity of time consuming serial passaging and associated cell sorting or enrichment, and simply, in one step, homogenous MSC-like cells were obtained. The spindle-shaped cells and their subcultures showed characteristic MSC surface markers including being positive for CD73, CD90, CD105, CD146 and CD166, and negative for hematopoietic markers CD34 and CD45. The cells also were capable of tri-lineage differentiation, indicating their multi-potency, as shown through *in vitro* studies. Thus the derived cells are referred to as MSC-like cells.

It is noteworthy that the MSC-like cells derived on the Type I collagen coating displayed a unique growth pattern over time, in that at 10 days the cells were found growing in isolated, separate colonies mainly located near the edge of the culture wells rather than growing as a uniform monolayer of cells. In contrast, on tissue culture treated polystyrene (i.e., the conventional culture surface), neither hESCs nor hiPSCs differentiated into spindle-shaped cells, although they did have more robust sustained attachment and growth than Type I collagen over a large area of the plate. It is not yet known why the cells on collagen preferentially colonized the edges better than other regions of the collagen-coated well although the collagen coating may be slightly thicker at the plate edges. Increasing the seeding density from 15,000 to 50,000/cm^2^ did not change the growth pattern in our study, i.e., by day 10 the cells repeatedly formed separate colonies on collagen instead of a continuous monolayer, implying other mechanisms than cell density were contributing to the unique growth pattern.

We did not yet identify the mechanisms underlying the enhanced derivation process on Type I collagen in the present study. However, other relevant studies provide clues that EMT might have played a fundamental role in the derivation process. EMT is known for its roles in embryogenesis, organ development, wound healing and tissue regeneration [28]. There is evidence that hESCs can develop an epithelial phenotype after monolayer culture or from migration out of embryoid bodies which then undergo EMT to acquire mesenchymal phenotype [7], [8]. In the present study, the single cells plated for 10 days on tissue culture plates did have epithelial cell-like characteristics such as close cell packing and tight junction-like structures (Figure 2B inset). Cells on collagen coatings did not show such an overt epithelial-like phenotype possibly due to their rapid transition to a mesenchymal phenotype stimulated by the fibrillar collagen coating. A rapid transition to MSC-like cells could also have arisen from a possible selection process whereby slightly differentiated cells with less-embryonic, more mature cell surface receptors were preferentially able to attach to the Type I collagen. Type I collagen is known to be able to stimulate and promote EMT through integrin-mediated signaling pathways [17]–[19] and retain osteogenic differentiation potential of human adult MSCs during ex vivo expansion [15]. This postulated mechanism could be addressed with the application of integrin-blocking antibodies and will be examined in future studies.

Dissociation of hESCs and hiPSCs into single cells was a necessary step in the present method as evidenced by the failure of the experiments in which traditional multi-cellular colonies were plated. Directly plating colonies of hESCs and hiPSCs, did not give rise to the spindle-shaped like cells on either the tissue culture polystyrene or the collagen coating. Instead, the cell colonies tended to develop into a mixture of various cell types judging from the heterogeneous cell morphologies. The non-dissociated colonies preserved the tight cell-cell interactions resembling the conditions for EB formation, which might have similarly favored spontaneous differentiation. We postulate that a separation of the cells from each other forced a direct interaction of the cell surface receptors with the substrate materials, rather than to each other, thereby enhancing the effect of the substrate in the differentiation process. The success of the biomaterials substrata to direct the differentiation process demonstrated here further strengthens the growing body of literature indicating that biomaterials, much like the extracellular matrix, can direct stem cell fate [10], [29].

Dexamethasone was present in the derivation medium, and might also have positively contributed to the mesenchymal differentiation. Dexamethasone is commonly used in osteogeneic, chondrogeneic and adipogenic cultures [7], [30] and is also used for promoting *in vitro* proliferation of MSCs by boosting the effects of other growth factors [31], [32]. The addition of dexamethasone might have expedited the differentiation process through enhancement of the activity of the growth factors produced by the cells themselves or present in the fetal bovine serum.

The tri-lineage differentiation potential of the derived MSC-like cells was demonstrated through *in vitro* studies. Chondrogenic differentiation was easily achieved as evidenced by the strong gene expression of cartilage matrix components including aggrecan and collagen type II and transcription factor SOX9. Histological examination displayed a prevalent distribution of acidic glycosaminoglycans in the pellet. Significant production of aggrecan and collagen type I was evidenced by immunohistochemical staining. The adipogenic culture conditions induced apparent lipid accumulation in the cytoplasm and adipocyte gene expression after 21 days, although PPARγ expression was unexpectedly low given the amount of lipid droplets in the cells. Osteogenic differentiation was demonstrated by mRNA level gene expression of RUNX2, COL1A1 and ALP. ALP was significantly up-regulated in response to exposure to osteogenic medium and resulted in mineral deposition. However, late stage osteoblast markers were not present in the cultures as evidenced by the lack of osteocalcin and bone sialoprotein gene expression (the C_t_ of real-time PCR reaction was more than 35). The inability of human marrow derived MSCs to mature into late-stage matrix producing osteoblasts *in vitro* has also been observed by other researchers [9], [33]. The similar expression of RUNX2 and COL1A1 mRNA before and after osteogenic differentiation observed in these MSC-like cells derived from human pluripotent stem cells is consistent with a previous observation during osteogenic differentiation of human bone marrow derived MSCs [34], [35]. Osteogenic differentiation of the MSCs can be associated with increases in DNA-binding activity of Runx2 without changes in mRNA level expression [34]. It should be noted that the differentiation potential of human adult MSCs is donor dependent [36], source tissue dependent [37] and that repeated passaging leads to a reduced osteogenic differentiation capacity [4]. Recently it was shown that only 2 of 10 donors had a strong chondrogenic potential [36]. Since there is no standard adult MSC cell source with a standard gene expression profile at a given time point, adult MSCs were not included as controls in these *in vitro* studies. Based on these promising *in vitro* assay results, *in vivo* differentiation studies should be pursued to conclusively demonstrate the multi-potentiality and lack of teratoma formation of these MSC-like cells.

In conclusion, a one-step MSC derivation method from pluripotent stem cells is reported in which singly dissociated H9 hESCs and YK26 iPSCs are cultured on biomimetic fibrillar Type I collagen coatings to obtain MSC-like cells. Both H9-hESCs and YK26-iPS cells responded similarly when subjected to the present MSC derivation process on fibrillar Type I collagen. The derived MSC-like cells from both cell lines displayed high expression levels of surface markers typical of MSCs, and the cells demonstrated *in vitro* tri-lineage differentiation potential including osteogenesis, chondrogenesis and adipogenesis. *In vivo* studies are needed to fully confirm the ability of these progenitors to function adequately in a therapeutic setting, nonetheless multipotent stem cells with properties resembling that of MSCs were successfully obtained. The biomimetic fibrillar type I collagen coating played a necessary role in the mesenchymal derivation as verified by the absence of the transition to MSC-like cells on controls of tissue culture treated polystyrene, although the detailed mechanism is not yet known. Most importantly, the new method presented is not only a convenient and efficient way to induce mesenchymal-like stem cells from hES and iPS cells, but also demonstrates the successful strategy of using an appropriate biomaterial matrix to influence and direct stem cell differentiation.

## Materials and Methods

### Preparation of biomimetic fibrillar Type I collagen coatings

Purified bovine Type I collagen monomer stabilized in an acidic solution (PureCol, Inamed Biomaterials, CA) was used at a concentration of 300 µg/ml to prepare biomimetic fibrillar collagen coatings following a protocol developed by the National Institute of Science and Technology (NIST) [27]. Briefly, the collagen was diluted into a phosphate buffered saline solution (PBS) consisting of 1× PBS: 0.1 M NaOH:10× PBS: 3 mg/ml collagen stock solution at a 70∶1∶1∶8 ratio (vol∶vol) in a 50 ml conical tube. The collagen stock solution was added last to minimize the formation of insoluble aggregates. One ml of the diluted solution was added per well in 12-well non-tissue culture plates. After incubation at 37°C in humidified air with 5% CO_2_ for 12–21 hours, the gel-like supernatant was aspirated from the plates and the coated surfaces were rinsed with PBS twice. 1 ml PBS was added to each well to cover the coatings. The coated plates were sealed and stored at 4°C for later use. The coating typically comprised of Type I collagen fibrils of ∼250 nm in thickness and several microns in length (Fig. 1).

### Culture of hESCs and iPSCs

H9 hESCs (WiCell, Madison, WI, USA) and human induced pluripotent stem cell line HDFa-YK26 (YK26-iPSCs; Stem Cells Core at UConn Health Center) [38] were maintained on Matrigel™ (BD Biosciences, Mountain View, CA) and fed with mouse embryonic fibroblast (MEF)-conditioned medium supplemented with 4 ng/ml bFGF (Invitrogen). The basal medium used for preparing the MEF-conditioned medium contained basal Dulbecco's modified Eagle's medium (DMEM)/F-12, 20% knockout serum replacement, 2 mM nonessential amino acids, 2 mM L-glutamine (all from Invitrogen, Carlsbad, CA), and 0.1 mM beta-mercaptoethanol (Sigma-Aldrich, St. Louis, MO). MEF cells were mitotically inactivated by gamma irradiation (irr-MEF) for producing the MEF-conditioned medium used to culture the hESCs and hiPSCs prior to the derivation step [39]. The maintenance medium was replenished daily for the hESCs and hiPSCs culture. H9 cells at passage 47 and YK26-iPSCs at passage 67 were used for the derivation.

### Derivation of MSC-like cells on fibrillar collagen coatings

Before dissociation, H9-hESCs and YK26-iPSCs, maintained on Matrigel™, were treated with 10 uM ROCK inhibitor Y-27632 in maintenance medium for 1 hour. Colonies were then dissociated into single cells after incubating with 1 ml Accutase® per well for 10 minutes in a 37°C incubator. The cells were spun down and re-suspended in the maintenance medium supplemented with 10 uM ROCK inhibitor Y-27632. The single cells were seeded onto the collagen coating at a density of 15,000/cm^2^ in the maintenance medium. After 24 hours, the maintenance medium was supplemented with an equal volume of the derivation medium which contained basal alpha-MEM (Invitrogen), 10% FBS (Hyclone), 100 U/ml penicillin and 100 ug/ml streptomycin (Invitrogen), 100 nM dexamethasone (Sigma-Aldrich) and 50 uM magnesium L-ascorbic acid phosphate (Sigma-Aldrich). After incubation for two more days, the medium was replenished with the derivation medium and changed every 3–4 days thereafter. At 10 days, the cells were harvested and labeled as passage zero (P0). The cells were expanded on new collagen coating using an expansion medium containing alpha-MEM (Invitrogen), 10% MSC-qualified FBS (Hyclone), 100 U/ml penicillin and 100 ug/ml streptomycin, 2 mM L-glutamine and 0.1 mM non-essential amino acid (all from Invitrogen). The expansion medium was replenished every 3–4 days. Cells were passaged upon subconfluency, usually within 5–7 days, at a 1∶3 split ratio. The second passage (P2) was used for the tri-lineage differentiation evaluation and flow cytometry analysis.

In control studies, the singly dissociated hESCs and iPSCs were plated onto tissue culture-treated polystyrene (conventional tissue culture plates) instead of the collagen coating, but otherwise with the same manipulation of the cells as described above. In other controls, hESC and iPSC colonies instead of dissociated cells were plated and cultured on both tissue culture plates and collagen coatings. In these studies, the cells were not treated with ROCK inhibitor Y-27632 since intact colonies of pluripotent cells survive well after passaging.

### Flow Cytometry

The cells derived on the collagen coatings were passaged on the collagen coating (P2), then the surface antigen profile was probed by flow cytometry. A panel of surface markers typical for characterizing human MSCs was used, including: CD73, CD90, CD105, CD146, CD166, CD34 and CD45 [8]. Surface antigen profiles were analyzed on a BD LSR II Flow Cytometer (Becton-Dickinson, San Jose, CA). Cells at or near confluence were harvested from the collagen coating using 0.05% Trysin-EDTA and washed twice in PBS containing 15% fetal bovine serum (FBS, Invitrogen). After filtering through a 70 um cell strainer, cells were spun down and then re-suspended in staining medium containing 1× HBSS, 10 mM HEPES at pH 7.4 and 2% FBS. Cells were separately labeled with fluorochrome-conjugated antibodies including phycoerythrin (PE) conjugated anti-human CD73 antibody (BD Pharmingen), FITC conjugated anti-human CD90 (Thy1) antibody (eBioscience), PE conjugated anti-human CD105 antibody (eBioscience), Alexa Fluor 647 conjugated anti-human CD146 (Biolegend), PE conjugated anti-human CD166 (Biolegend), FITC conjugated anti-human Stro-1 (Biolegend), FITC conjugated anti-human CD34 antibody (BD Pharmingen), Pacific Blue (PB) conjugated anti-human CD45 antibody (eBioscience). After a 45 minute incubation, cells were washed twice with staining buffer. Unstained cells were used as a control. At least 10,000 events were acquired for each sample. The data were analyzed using FlowJo software (Tree Star Inc, Ashland, OR).

### Tri-lineage differentiation

The functional differentiation ability of the derived MSC-like cells was tested *in vitro*. The osteogenic medium contained alpha-MEM (Invitrogen), 10% FBS (Hyclone), 100 U/mlpenicillin and 100 ug/ml streptomycin (Invitrogen), 100 nM dexamethasone (Sigma-Aldrich), 50 uM magnisum L-ascorbic acid-phosphate (Sigma-Aldrich), and 4 mM beta-glycerophosphate (Sigma-Aldrich). Cells were seeded at density of 30,000/cm^2^ in 12 well tissue culture plates (Falcon, Becton-Dickinson, NJ). Three replicates were analyzed for each assay. The medium was replenished every 3–4 days. Mineral deposition in the osteogenic cultures was detected by staining with 20 ug/ml xylenol orange (XO) supplemented to the culture medium 24 hours before analysis. XO is a fluorescent dye that has been used for labeling of calcium phosphate mineral deposition in osteogenic cultures [40]. Positive staining was visualized using fluorescence microscopy.

Chondrogenic differentiation assays were performed using a standard pellet culture method [41]. The chondrogenic medium contained high glucose DMEM (Invitrogen), 1% ITS+Premix (Becton-Dickinson), 100 mM sodium pyruvate (Invitrogen), 10 ng/ml TGF- β1 (Peprotech), 100 nM dexamethasone (Sigma-Aldrich), 120 uM magnesium L-ascorbic acid-phosphate (Sigma-Aldrich), and 100 U/ml penicillin and 100 ug/ml streptomycin (Sigma-Aldrich). The pellets were formed in 5 ml polypropylene tubes (Becton-Dickinson) by centrifugation of 200,000 cells at 600×g for 5 min. Three replicates were analyzed for each assay. Medium was changed every 3–4 days. Histological examination was performed on frozen sections of the chondrogenic pellets. Pellets were fixed in 10% neutral buffered formalin. The sections were prepared by embedding the fixed pellets in OCT (Tissue-Tek, Ted Pella Inc, CA) and 7 um sections were made using a Cryostat (Leica Microsystems Inc, Bannockburn, IL). Sections were stained with alcian blue solution which was prepared by adding 1 g alcian blue 8 GX (Sigma-Aldrich) to 100 ml 3% acetic acid solution at pH 2.5. The sections or pellets were soaked in alcian blue for 30 minutes, followed by rinsing with pH 1.0 acetic acid once and pH 2.5 acetic acid twice. For immunohistochemical analysis, sections were treated for 30 min at 37°C in 0.05% Trypsin (Invitrogen). Endogenous peroxidase activity was quenched by incubation with 3% hydrogen peroxide (Sigma-Aldrich) for 10 minutes at room temperature. Primary antibodies raised against aggrecan (cat# sc-16492) and COL1A1 (cat# sc-28887) (both from Santa Cruz Biotechnology Inc, CA) were added at 1∶50 dilution and incubated for 1 hour at 37°C. The biotin conjugated secondary antibodies recognizing immunoglobin of either goat (Cat#305-065-045) or rabbit (Cat#111-065-003) (both are from Jackson ImmunoResearch Laboratories Inc, West Grove, PA) were added at 1∶1000 dilution and incubated for 1 hour at 37°C. Streptavidin-conjugated horseradish peroxidase (HRP) from an ultra-sensitive ABC peroxidase staining kit (Pierce, Rockford, IL) was added to react with the biotin conjugated secondary antibody for 30 minutes at 37°C, then a diaminobenzidine (DAB) chromogenic subtrate kit (Pierce, Rockford, IL) was used to develop the staining.

The adipogenic differentiation medium consisted of high glucose DMEM (Invitrogen) supplemented with 10% FBS (Hyclone), 1 uM dexamethasone (Sigma-Aldrich), 100 uM indomethacin (Sigma-Aldrich), 500 uM 3-isobutyl-1-methylxanthin (IBMX) (Sigma-Aldrich), and 10 ug/ml insulin (Sigma-Aldrich), and 1% penicillin and streptamycin (Invitrogen). Cells were seeded at a density of 15,000/cm^2^ in 12 well tissue culture plates (Falcon, Becton-Dickinson). Medium was replenished very 3–4 days. Lipid production in the 21 day adipogenic cultures was examined with Oil Red O (ORO) staining. The staining solution was prepared by dissolving 0.7 g Oil Red powder (Sigma) in 200 ml of isopropanol (Sigma-Aldrich), followed by dilution with distilled water at a ratio of 3∶2 (3 parts Oil Red O stock: 2 parts distilled water). Cells were fixed with 10% formalin for 5 minutes at room temperature, then stained with Oil Red-O for approximately 10 minutes, followed by rinsing once with 60% isopropanol and then water.

### Reverse Transcription-quantitative Polymerase Chain Reaction (RT-qPCR)

RNA was extracted using Trizol (Invitrogen) according to the manufacturer. Genomic DNA was removed by treatment with DNase I following the manufacturer manual (Invitrogen). Total 10 µg of RNA was used for cDNA synthesis using the High Capacity cDNA Reverse Transcription Kit (Applied Biosystems, Foster City, CA) performed with the iCycler® Thermal Cycler (Bio-Rad Laboratories, Richard, CA) following the manufacturer's instructions. TaqMan Gene Expression Assays for RUNX2 (Hs00231692_m1), COL1A1 (Hs00164004_m1), ALP (Hs00758162_m1), OC (Hs01587813_g1), BSP (Hs00913377_m1), SOX9 (Hs00165814_m1), COL2A1 (Hs00264051_m1), ACAN (Hs00153936_m1), COL10A1 (Hs00166657_m1) and GAPDH (Hs99999905_m1) were purchased from Applied Biosystems. Quantitative RT-PCR reactions were carried out on an ABI HT7900 system (Applied Biosystems) following the manufacturer's protocol. Quantification was performed with the Delta-Delta Ct method using GAPDH as an internal control.

For adipogenic gene analysis quantitative PCR was carried out using the SYBR Green PCR master mix platform (ABI). Primer pairs for GAPDH (GCACCGTCAAGGCTGAGAAC; ATGGTGGTGAAGACGCCAGT) and PPARγ (TGCCAAAAGCATTCCTGGTT; TCGCTGTCATCTAATTCCAGTGC) [42] and FABP4 (ACTGGGCCAGGAATTTGACG; CCCCATCTAAGGTTATGGTGCTC) retrieved from PrimerBank [43] (PrimerBank ID: 168480125b1) were used.
